# Single-centre, randomised clinical trial of the immunomodulatory mechanisms of daily supplementation of palm tocotrienol-rich fraction in healthy human volunteers following influenza vaccination

**DOI:** 10.12688/f1000research.137005.1

**Published:** 2024-02-22

**Authors:** Ammu Kutty Radhakrishnan, Badariah Ahmad, Kanga Rani Selvaduray, Sitti Rahma Abdul Hafid, Uma Devi Palanisamy, Cheng Zsien Zhin

**Affiliations:** 1 Jeffrey Cheah School of Medicine and Health Sciences, Monash University Malaysia, Sunway, Selangor, 47500, Malaysia; 2 Product Development and Advisory Services, Malaysian Palm Oil Board, Bandar Baru Bangi, Selangor, 43650, Malaysia; 3 Hovid (M) Ltd, Ipoh, Perak, Malaysia

**Keywords:** tocotrienols, tocotrienol-rich fraction (TRF), influenza vaccine, immune response

## Abstract

**Background:**

Vitamin E from palm oil, known as the tocotrienol-rich fraction (TRF), has been shown to have immune-enhancing activity. To date, only one dose of TRF (400 mg daily) has been tested in a clinical trial. The proposed study will evaluate the immune-enhancing activity effects of lower doses (200, 100 and 50 mg) in a clinical trial using an influenza vaccine as the immunological challenge.

**Methods:**

A single-centre, randomised, parallel, double-blinded, placebo-controlled clinical trial with balance allocation involving five arms will be conducted. The healthy volunteers recruited will be randomly assigned to one of the arms, and they will be asked to take the respective supplements (400 mg, 200 mg, 100 mg, 50 mg of TRF or placebo) daily with their dinner. The volunteers will receive the influenza vaccine after four weeks. They will be asked to return to the study site four weeks later. A blood sample will be taken for the study at baseline, four and eight weeks. Primary outcome measures will be antibody levels to influenza, blood leucocyte profile and cytokine production. Secondary outcomes will be correlating plasma vitamin E levels with immune responses, plasma proteins and gene expression patterns. The findings from this study will be published in relevant peer-reviewed journals and presented at relevant national and international scientific meetings.

**Conclusions:**

The recent world events have created the awareness of having a healthy and functional immune system. Nutrition plays an important role in helping the immune system to function optimally. This study will show the effects of lower doses of TRF in boosting the immune response of healthy individuals and also elucidate the mechanisms through which TRF exerts its immune-enhancing effects.

**Clinical trial registration:**

Australian New Zealand Clinical Trials Registry (ANZCTR) [ ACTRN12622000844741] dated 15 June 2022.

**Protocol version:**

2

## Introduction

The host immune system plays a pivotal role in protecting the body from infectious agents and cancers. ^1^^–^^4^ Appropriate and effective immune responses are essential to distinguish foreign antigens, such as infectious agents (e.g. viruses, bacteria, parasites and fungi) and abnormal or malignant cells from normal “self” antigens to prevent unwanted immune responses. The immune system is characterised by two mechanisms, the innate and the adaptive immune systems. The innate immune system comprising monocytes, macrophages, neutrophils, natural killer cells, dendritic cells, and granulocytes serves as the first line of defence against foreign antigens, directly targeting and facilitating the activation of the adaptive immune system. ^5^^–^^7^ The T- and B-lymphocytes are the key players of the adaptive immune system. The lymphocytes are not the first line of defence against foreign antigens as these cells need to be appropriately activated upon antigen recognition. Once activated, the lymphocytes elicit a more efficient and specific immune response as well as develop immunological memory. ^6^^,^^8^^,^^9^

A well-balanced nutritional status is essential for maintaining normal immune function. Deficiencies in nutrients often lead to impaired immune system function; on the contrary, intake at suggested levels can lead to normal or enhanced immune function. ^2^^,^^3^^,^^10^ Nutritional interventions have powerful effects on activating the host immune system. ^10^^–^^12^ One compound is vitamin E, which consists of tocopherols and tocotrienols. The vitamin E from palm oil, known as the tocotrienol-rich fraction (TRF), is a mixture of tocotrienols (70%) and tocopherols (30%). ^13^^,^^14^ Several clinical and experimental model-based studies have shown that tocotrienols have several biological activities such as antioxidant, ^15^^,^^16^ lipid-lowering, ^17^^,^^18^ immune-enhancing, ^19^^–^^21^ anticancer, ^22^^–^^24^ neuroprotective, ^25^^–^^27^ and anti-diabetic. ^28^^,^^29^ Tocotrienols may cause these effects by acting at the epigenome, genome or protein levels. ^30^ Recently, it was reported that consuming TRF can also cause changes to the gut microbiome. ^31^^,^^32^

In an earlier study, we reported that daily supplementation of 200 mg TRF or α-tocopherol (α Toc) did not induce significant immunomodulatory changes in healthy human volunteers in the absence of any immunological challenge. ^33^ In a subsequent study, we included an immunological challenge in the form of tetanus toxoid (TT) vaccination in healthy individuals supplemented daily with 400 mg TRF. Daily supplementation of 400 mg TRF produced a significantly higher immune response to the TT vaccine. ^19^ A recent report showed that daily supplementation of 150 mg TRF for six months modulated some genes related to the immune system in healthy older adults. ^34^ In the literature, most studies have used a single dose of TRF to report their findings. To date, no studies have evaluated the dose-response effects of TRF supplementation on the host immune system. So, there is a need to evaluate the effectiveness of supplementing daily with lower doses of TRF (200, 100, or 50 mg) to boost immune response following an immunological challenge such as a vaccine. The influenza vaccine was chosen in this study as the vaccine model, as flu is a major problem faced by individuals worldwide.

## Objectives

This study aims to investigate the effects of different concentrations of TRF (400, 200, 100 or 50 mg) on the immune response to an influenza vaccine in healthy individuals and to elucidate the mechanism of action. Using high and low dosages is important to understand what may happen following TRF supplementation. This information can be used to develop this in a range of functionality. The specific objectives are (i) to compare antibody responses to influenza antigen following daily supplementation with TRF (400, 200, 100 or 50 mg) with placebo; (ii) to compare leucocyte profiles and cytokine levels in response to influenza antigen following daily supplementation with TRF (400, 200, 100 or 50 mg) with placebo; (iii) to identify differentially expressed plasma proteins following daily supplementation with TRF (400, 200, 100 or 50 mg) with placebo in the presence or absence of vaccination; and (iv) to correlate plasma vitamin E levels with antibody levels in volunteers with and without daily supplementation with TRF.

### Research question

Will daily supplementation of lower doses of TRF (400, 200, 100 or 50 mg) enhance the immune response to an immunological challenge such as the influenza vaccine?

### Study hypothesis

The research hypothesis of this study is that daily supplementation of lower doses of TRF will enhance the host’s immune response to an influenza vaccine.

### Outcomes

The primary outcomes of this trial will be comparing the antibody levels to influenza, the blood leucocyte profile, plasma vitamin E and cytokine production between the different groups. Secondary outcomes will be correlating plasma vitamin E levels with immune responses, plasma proteins and gene expression patterns.

## Protocol

### Ethics consideration

The Monash University Human Ethics Committee (MUHREC) have reviewed and approved the study protocol [MUHREC 30401] (27 January 2022). In addition, the study will be conducted in accordance with the principles of the Declaration of Helsinki 2013 and the Malaysian laws on medical research involving human subjects. All changes to changes to the protocol will require approval from MUHREC before they are applied. All data, including personal information about potential and enrolled participants for this study, will be stored in a file kept in a locked cabinet in a locked room in Monash facilities. The digital data will be stored on a secure shared drive behind the Monash firewall. Only the chief investigator will have access to these files. Upon completion of the study, all data will be stored and handled by Monash University Malaysia for at least 15 years after the study has ended. Only researchers listed in the ethics approval will have access to the research data.

This protocol has been registered with the Australian New Zealand Clinical Trials Registry (ANZCTR) [ ACTRN12622000844741] on 15 June 2022.

### Trial design

A single-centre, parallel, double-blinded, placebo-controlled with balanced randomisation into five arms ( Table 1).

**Table 1.** Study groups.

Group	Daily supplementation (Day 0 to Day 56)	Number of volunteers	Influenza vaccine
A	Placebo (0 mg TRF)	30	Day 28
B	50 mg TRF	30	Day 28
C	100 mg TRF	30	Day 28
D	200 mg TRF	30	Day 28
E	400 mg TRF	30	Day 28
	Total volunteers	150	

TRF = Tocotrienol-rich fraction.

### Sample size

The sample size was calculated using G*Power version 3.1.9.4. With the power set at 80% and a 5% level of significance, the analysis showed that 26 subjects per arm were required to detect a large difference between groups. The effect size (d) was assumed to be 0.7, based on a previous study. ^19^ Assuming a 15% dropout rate in two months, 30 subjects per arm (150 subjects) will be enrolled.

### Participants

This study will be conducted at the Thomson Hospital, Kota Damansara, Malaysia. Thomson Hospital is a private tertiary-level health facility in Kota Damansara in Selangor, Malaysia. Participants will be invited to join this study by way of placing posters around the hospital’s primary care clinics and through social media. Hence, some of the volunteers recruited will include healthy hospital employees and their relatives, who became aware of the study through the posters placed in the hospital. Volunteers will also recruited using social media (Facebook, Instagram and WhatsApp).

### Study plan

Healthy subjects (n = 200-250) aged between 25 to 60 years old will be invited to participate in this study. At the time of screening, the purpose of the study and what the study entails will be explained to all volunteers who express an interest in joining the study. Before recruiting a subject for this study, a blood sample (12 mL) will be taken from all interested volunteers, where 10 mL of the blood will be sent to the Thomson Hospital medical diagnostic laboratory for various biochemical tests to establish baseline parameters to assist in the selection of healthy volunteers. The remaining 2 mL of blood will be used to quantify baseline plasma levels of vitamin E and anti-influenza antibodies. Then, 150 healthy volunteers who meet the inclusion and exclusion criteria ( Table 2) of this study, with the lowest level of anti-flu antibody and low plasma vitamin E, will be recruited for the study. Once a volunteer is selected for the study, a physician will explain the study protocol to the volunteer and ask the volunteer to provide a signed written informed consent to participate in this study.

**Table 2.** Inclusion and exclusion criteria.

Inclusion criteria	Exclusion criteria
• 18–60 years-old • Healthy and free of diseases *(biochemical tests)*	• Have some health-related problems • On treatment or medication • Currently taking vitamin E supplements • Smoker • Received influenza A and B vaccination in the last 12 months • Obese (BMI > 35 kg/m ^2^) • Pregnant and/or breast-feeding

### Study approach

**Figure 1.**
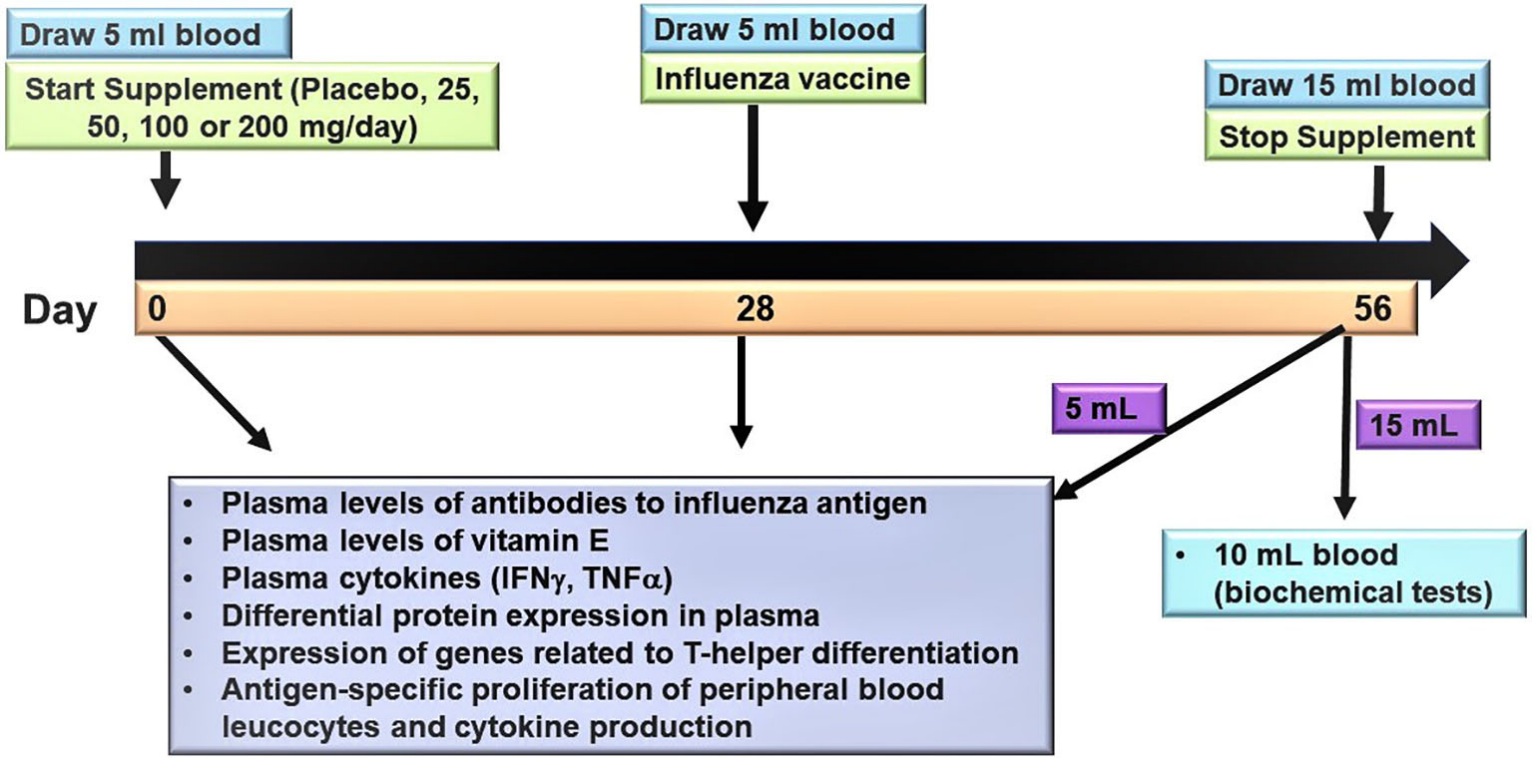
Overview of the study approach.

The study is expected to screen around 200–250 volunteers to recruit 150 volunteers required for this study. During the screening step, the volunteers will be subjected to a routine simple medical history and health screening to collect some data [age, gender, eating habits, occupation, lifestyle (smoking, alcohol, exercise), height, weight] as well as biochemical blood [full blood count (FBC), glucose, HBA1c, lipid profile, estimated glomerular filtration rate (EGFR), liver function tests (LFT)] and urine [full examination microscopy examination (FEME)] tests. Once the healthy volunteers are selected, they will be randomly assigned to one of the five study groups ( Table 1). The volunteers will be asked to attend three clinic sessions (days 0, 28 and 56) ( Figure 1). A trained phlebotomist will take blood via venepuncture, as shown in Figure 1. After the blood-taking on day 0, the volunteers will be given a bottle containing sufficient test supplements for 28 days. The volunteers will be asked to take the supplement after dinner every day for 28 days.

The volunteers will be asked to return to the study site on day 28 for blood-taking (5 mL) and to receive an influenza vaccine. The volunteers will be given a second bottle containing sufficient supplements for another 28 days. The volunteers will return to the study site on day 56 for blood-taking (15 mL) ( Figure 1). Of the 15 mL of blood taken on Day 56, 10 mL will be sent to the Thomson Hospital medical diagnostic laboratory for various biochemical tests to establish the end-study parameters. At the second (day 28) and third (day 56) clinic visits, the volunteers will be asked to return the bottle with their supplement. The pill count will check compliance at each visit. In addition, the volunteers will be asked to maintain a normal balanced diet and exercise/activity regimen during the study period. They will be asked to keep a diary to record major deviations from their diet. The diary will be checked at each visit for any unusual entries.

### Intervention

The TRF used in this study will be Tocovid Suprabio ^TM^, manufactured by HOVID Ltd in Malaysia, and is a registered supplement in Malaysia that is sold as an over the counter (OTC) supplement in major pharmacies in Malaysia. The active ingredient of the Tocovid SupraBio ^TM^, called EVNol ^TM^ was manufactured by ExcelVite Sdn. Bhd., Malaysia. The TRF has received the generally regarded as safe (GRAS) status by the USA Food Development Authority (FDA). ^35^

The influenza vaccine used in this study will be the VaxigripTetra Influenza vaccine type A and B as per the WHO recommendations, which will be obtained from the Thomson Hospital’s pharmacy.

### Blinding

The supplements will be packed in identical bottles and packaging. Each package will be labelled anonymously with a running number. The supplements in the first four weeks will be labelled with a number and an additional label, “FIRST”, while supplements in the second four weeks will have the same number, but the additional label will be “SECOND”. All investigators and volunteers will be blinded to the intervention. Then, a simple randomisation process allocated the intervention package to the volunteers, where the first volunteer recruited will receive the first anonymous number with the label, “FIRST” while the second volunteer recruited will receive the second anonymous number with the label, “FIRST” and so on for their first four weeks of supplementation. When they return to the study site after four weeks, they will receive a supplement package having the same number as their first package but with the label, “SECOND”. Each subject will receive the package with the supplement having a number from the study nurse. The number on each bottle will correspond to a supplement listed in Table 1, which only the principal investigator of the study will know. All other investigators in the study will be blinded to this information.

Unblinding will only occur at the end of the study after all data have been collected for analysis. However, if any of the subjects in the study were to experience any adverse effects, then unblinding will be permitted for the said volunteer for further action.

### Criteria for discontinuation

TRF has received the GRAS status from the US FDA. ^35^ Hence, severe adverse effects following the consumption of TRF are not expected. However, volunteers can drop out of the study at any time if they experience any side effects or do not want to continue with the study.

### Laboratory tests

*Antibodies to human influenza viruses*

The level of antibodies to influenza antigen in the plasma taken on days 0, 28 and 56 will be determined using an enzyme-linked immunosorbent assay (ELISA). Briefly, 100 μL of the flu vaccine (1 μg/mL) in coating buffer (Thermo Fischer Scientific, USA) will be added to 96-well ELISA plates (Corning C62-9018 plate) and incubated at 4°C for 24 hours. The plate will be washed thrice with 250 μL of ELISA wash buffer (Thermo Fischer Scientific, USA). Then, the plate will be blocked with 250 μL of ELISA diluent (Thermo Fischer Scientifc, USA) at room temperature for 2 hours. The plate will be washed thrice with 250 μL of ELISA wash buffer (Thermo Fischer Scientific, USA). The plate will be dried by gently tapping on absorbent paper. Then, 100 μL of diluted human serum (baseline, Day 28 and Day 56) will be added to each well in duplicates. A standard curve of anti-influenza A (Santa Cruz, USA) and anti-influenza B (Santa Cruz, USA) antibodies will be used to quantify anti-influenza A and anti-influenza B levels in the serum of healthy volunteers at different time points. Blank wells will contain 100 μL of ELISA diluent (Thermo Fischer Scientific, USA). After 2 hours at room temperature, the plate will be washed five times with 250 μL of ELISA wash buffer (Thermo Fischer Scientific, USA). Then, 100 μL of anti-human Ig (IgG, IgM and IgA) or anti-human IgG conjugated with horseradish peroxidase (HRP) (Abcam, USA) will be added to all wells. After one hour at room temperature, the plate will be washed five times with 250 μL of ELISA wash buffer (Thermo Fischer Scientific, USA), and 100 μL of TMB ELISA substrate (Abcam, UK) will be added to all wells. The enzymic reaction will be stopped after 10-20 mins by adding 100 μL Stop solution for TMB substrate (Abcam, UK). Absorbance at 450 nm will be read using a microplate reader (Tecan, Switzerland).

*Antigen-specific proliferation*

The buffy coat will be isolated from peripheral blood and cultured in the presence of the influenza vaccine for 72 hours. The antigen-specific proliferation will be measured using a commercial cell proliferation kit. The culture supernatant will be harvested and stored at -80°C until used for cytokine analysis.

*Plasma levels of vitamin E*

Plasma levels of vitamin E will be quantified from samples taken on days 0, 28 and 56 using the high-performance liquid chromatography (HPLC) [Agilent HPLC 1200 with fluorescence detector, Agilent, USA] as desbribed previously. ^36^ Briefly, 1.0 mL of 0.9% sodium chloride (NaCl) will be added to 1.0 mL of human plasma in a glass test tube and vortexed for 30 seconds. Then, 1.0 mL ethanol-BHT (0.625 mg/mL) will be added, and the mixture will be vortexed for 30 seconds. Following this, 5.0 mL of n-hexane-BHT (0.05 mg/mL) will be added, and the mixture will be shaken overnight for 16 hours at 250 rpm, at 10°C using a shaking incubator (WIS-20, Witeg, Germany). After centrifugation (2500 rpm for 15 minutes at 4°C), the upper organic layer will be carefully extracted and transferred to a sterile tube. The tubes will be evaporated to dryness using a speed vacuum concentrator with CoolSafe -110°C cold trap (MaxiVac, LaboGene, Denmark). To obtain a standard curve for quantification, standard tocopherols (Toc) and tocotrienols (T3) (Hovid, Malaysia) will be separated on a Phenomenex KinetexTM PFP column (5.0 μm, 150 × 4.6 mm; Phenomenex) with a guard column using MeOH/H _2_O (87:13) as an eluent at a flow rate of 0.9 mL/min. The dried samples will be reconstituted with 0.3 mL of mobile phase [methanol (MeOH) + butylated hydroxytoluene (BHT) (0.625 mg/mL)] and filtered using a nylon syringe fitted with a 0.45 μm, 4 mm filter (Chrominex, Singapore) prior to HPLC analysis. Then, 10 μL of the preparation will be injected into the HPLC (Agilent HPLC 1200, USA). The fluorescence detector will be set an excitation wavelength of 296 nm and emission wavelength of 325 nm and photomultiplier tube (PMT) gain will be set at 10.

*Cytokines*

Plasma and culture supernatant stored at -80°C will be thawed and used to quantify plasma levels of cytokines [e.g. interferon-gamma (IFN-γ) (MABtech, USA), tumour necrosis factor-alpha (TNFα) (MABtech, USA), interleukin-12 (IL-12) (MABtech, USA), and interleukin-17A (IL-17A) (MABtech, USA)] using commercial ELISA kits.

*Protein expression*

The plasma protein concentrations will be determined using a commercial Bradford test. The plasma will be subjected to albumin and immunoglobulin elimination steps using the Pierce albumin/IgG removal kit (ThermoScientific, USA). Then, the protein will be processed using the EasyPep™ Mini MS Sample Prep Kit (ThermoScientific, USA) before being analysed with liquid chromatography-mass spectrometry/mass spectrometry (LCMS/MS) (Agilent, USA) to identify differentially expressed proteins (DEP). The expression of the selected DEP will be verified using ELISA or Western blotting.

*Gene expression studies*

Total RNA will be extracted from 1.5 mL of freshly drawn blood using a commercial ribonucleic acid (RNA) extraction kit (QIAGEN ^®^ RNA Blood Mini kit, QIAGEN GmBH, Hilden, Germany). Briefly, the red blood cells (RBC) will be lysed by adding 7.5 mL RBC lysis buffer (provided with RNA extraction kit, (QIAGEN, Germany). The mixture will be incubated on ice for 15 min, and the buffy coat will be recovered by centrifugation (10 min at 4°C). The supernatant will be discarded, and the pellet will be washed twice with 3 mL of the RBC lysis buffer and recovered by centrifugation (10 min at 4°C). Then, RNA will be extracted using RNA extraction, as recommended by the manufacturer (QIAGEN, Germany). The extracted RNA’s purity, quality, and integrity will be assessed using NanoDrop ^®^ ND-1000 (NanoDrop Technologies Wilmington, DE, USA) and Agilent 2100 Bioanalyzer (Agilent Technologies Santa Clara, CA, USA). The primers related to various genes associated with T-helper (Th) and T-regulatory (Treg) cells ( *Hoxa10, GATA3, RORα, CCR5, CXCR3 CCR7, IL-24, IL-12β2R, FoxP3; CD49b, folate receptor-4*) will be synthesised commercially (IDT Integrated DNA Technologies, Singapore). Quantitative PCR (QPCR) will be performed to compare mRNA expression between the groups and within the group (day 0 and day 56). The purified RNA will be processed using qPCRBIO SyGreen 1-Step Detect LOW-ROX kit (CAT#PB25.11-01), (QIAGEN, Hilden, Germany) for complementary DNA (cDNA) synthesis according to the manufacturer’s instruction. The total reaction volume per sample will be 20 μL with 10 ng of RNA template, 2 ng/μl of forward and reverse primers. Each sample will be analysed using commercial primer sets various genes associated with T-helper (Th) and T-regulatory (Treg) cells (Hoxa10, GATA3, RORα, CCR5, CXCR3 CCR7, IL-24, IL-12β2R, FoxP3; CD49b, folate receptor-4), which will be synthesized commercially (IDT Integrated DNA Technologies, Singapore). The GAPDH gene will be used as the housekeeping gene to normalize the quantitative polymerase chain reaction (QPCR) data. The cycling protocol will consist of 40 cycles involving denaturation at 95° for 5 seconds, followed by annealing and extension- steps, each at 60°C for 30 seconds. All samples will be run in triplicates. At the end of the 40 cycles, a dissociation curve analysis will be performed to confirm amplification specificity using the optical module PCR detection system available in the thermocycler (QuantStudio™ 5 Real-Time PCR System, Applied Biosystem, Thermo Scientific, USA). The ratio of gene expression of the target gene was compared between undifferentiated and differentiated cells using the formulae: 2-(ΔCt (test gene) – ΔCt (GAPDH)), where ΔCt represents the difference of threshold cycle (Ct) for each target gene.

### Statistical analysis

Statistical Package for the Social Sciences ( SPSS) version 18 will be used for the statistical analysis. The mean and standard deviation values will be calculated for each parameter and compared with the baseline and/or placebo. Differences between the baseline and/or placebo will be tested using a paired-t-test when data are normally distributed or Wilcoxon signed-rank when the data are skewed. The significance level will be set as a P value of ≤ 0.05.

### Dissemination

The findings from this clinical trial will be published in suitable peer-reviewed journals.

### Study status

This study is at the screening of volunteers’ stage to identify suitable subjects to be included in the study as per the inclusion and exclusion criteria ( Table 2) of this study.

## Discussion

At the start of 2020, a global public health challenge was caused by a novel coronavirus known as the severe acute respiratory syndrome coronavirus 2 (SARS-CoV-2). ^37^ This virus was named the novel coronavirus 2019 or COVID-19. COVID-19 was declared a pandemic by the World Health Organization (WHO) in March 2021. The pandemic has increased awareness of the need for a well-functioning immune system. ^38^^,^^39^ Nutritional interventions can potentially affect the host immune systems. ^9^^–^^10^ One such compound is vitamin E from palm oil. The vitamin E from palm oil is known as the tocotrienol-rich fraction (TRF), which contains tocotrienols (70%) and tocopherols (30%). ^13^^,^^14^ Several researchers have reported on the ability of TRF to modulate the host immune system. To the best of our knowledge, no studies investigated a dose-response curve using TRF supplementation and its impact on the host immune system. Previous studies have used high TRF concentrations, which may not be necessary to achieve the immune-enhancing effects in healthy individuals.

Furthermore, not many people may be able to afford a high dose of TRF. For any supplement, it is crucial to know what is happening biochemically at different dosages to understand the targeted effects as well as any side effects. It is important to use both high and low dosages in a study to fully understand what is happening in response to the intervention and develop this in a range of functionality. The influenza vaccine was chosen in this study as the vaccine model, as flu is a major problem faced by individuals worldwide.

## Data Availability

No data are associated with this article.
