# Supplementary material for: Single-centre, randomised clinical trial of the immunomodulatory mechanisms of daily supplementation of palm tocotrienol-rich fraction in healthy human volunteers following influenza vaccination
Source: F1000Res. 2024 Feb 22;13:135. [Version 1] doi: 10.12688/f1000research.137005.1 (PMC11391185; doi:10.12688/f1000research.137005.1)
Supplement: Peer review history

# Reviewer report: Reviewer response for version 1

**DOI:** 10.5256/f1000research.150153.r274149

**Published:** 2024-09-11

**Authors:** Pablo Cañete, Emely Hernandez Mateo

**Affiliations:** 1 The University of Queensland, Saint Lucia, Queensland, Australia; 2 Centre for Children’s Health Research, The University of Queensland, Saint Lucia, Queensland, Australia

Radhakrishnan and colleagues have designed a clinical study to interrogate the effects of tocotrienol-rich fraction (TRF) supplementation on influenza vaccine outcomes. Their protocol’s rationale is grounded on previous research that showed elevated antibody production in a tetanus toxoid immunisation study. The research is significant and timely, given the growing interest in nutritional interventions to boost immunity and particularly due to the ease of an oral administration treatment in the context of recent global health challenges like the COVID-19 pandemic. The study protocol has a strong design methodology as a randomized, double-blinded, placebo-controlled trial. The primary aim is to evaluate the immune-enhancing effects of different doses of TRF (50, 100, 200, and 400 mg) compared to a placebo, using an influenza vaccine as the immunological challenge, and by measuring several parameters indicative of immune function (antibody production, cytokine release upon rechallenge and soluble factors in the serum). While the study is interesting and will generate much-needed knowledge to mitigate poor vaccine outcomes, there are few concerns that need attention.

**Major concerns**

 The authors plan to gauge vaccine efficacy on the basis of, i) antigen specific antibodies, ii) soluble factors in the serum indicative of immune activity, and iii) cytokines produced by antigen-activated peripheral leukocytes. While all these metrics are indicative of immune activity, and thus suggestive of potential changes in vaccine efficacy, they are insufficient to definitively determine whether FRT enhances influenza vaccine outcomes for the following reasons:

1. High affinity antibodies are crucial for generating long-lived humoral memory in the form of a continuous secretion by long-lived plasma cells in the bone marrow. These antibodies have undergone affinity maturation in germinal centres, are mutated, and have such remarkable affinity that only minute amounts of it are needed to prevent re-infection. They form the foundation of most successful vaccines. Therefore, the authors need to measure antibody affinity to substantiate their conclusions regarding whether TRF enhances the quality of the immune response.
2. The ultimate metric for vaccine efficacy is protection: the proportion of individuals who are protected, develop mild disease, or remain asymptomatic upon re-exposure. I understand these exercises are difficult with, i) such low sample sizes, and ii) the likely budgetary and time constrains. ****However, the authors should at least try to address the previous point.
3. The sample size calculation accounts for a 15% dropout rate. However, there seems to be a discrepancy in the final number. If 26 subjects per arm are needed and the expected dropout rate is 15%, the correct sample size per arm should be: *Required per arm =* *26 / (* *1-0.15)* *≈30.6*. The result 30.6 should be rounded up to 31, not rounded down to 30. Sample size calculations should always be rounded up, not down, to ensure that the sample size is large enough. The reason for this is that the result represents the smallest sample size needed to produce the desired error. Thus, the total should be 31 subjects per arm for a total of 155 subjects (not 150). I recommend that the protocol manuscript is amended to increase the sample size to 155.

**Minor concerns**

- Readability is a minor issue for some sections of the manuscript. For example, the second paragraph of the blinding section is a bit confusing. There is also some repetition of the same sentences within the manuscript, for example the last paragraph of the introduction and the last paragraph of the discussion end with exactly the same sentence: “ *The influenza vaccine was chosen in this study as the vaccine model, as flu is a major problem faced by individuals worldwide”*. I recommend that all sections are reviewed for readability and consistence within the manuscript.
- FBC is not generally considered a biochemical test and should be classified as a haematological test (study approach section).
- Inconsistencies between information shown in protocol and in study registration website regarding data sharing:

The study registration entry in ANZCTR states that “De-identified raw data for each participant outside of the investigator team for the purposes of IPD meta-analyses, upon request” while the protocol says that “Only researchers listed in the ethics approval will have access to the research data.” The manuscript should specify the potential for data to be shared and the procedures for de-identification in the protocol.

**Blinding methodology: ** The blinding methodology seems appropriate; however, the manuscript would benefit from some clarifications:

-
  - Randomization Process: The phrase "a simple randomisation process" is vague. I recommend for the manuscript to describe the exact method used for randomization (e.g., computer-generated random numbers, block randomization, stratified randomization).

-
  - Allocation Concealment: The manuscript should state how the randomization list is generated and how allocation concealment is maintained. This section should include information specifying who generates the list and how it is kept from investigators and participants.

-
  - Blinding of Study Nurse: The manuscript should explicitly state if the study nurse is being blinded.

**Recommendation**

 Should the authors be willing to address the concerns outlined above, the article can be indexed.

Is the study design appropriate for the research question?

Partly

Is the rationale for, and objectives of, the study clearly described?

Yes

Are sufficient details of the methods provided to allow replication by others?

Partly

Are the datasets clearly presented in a useable and accessible format?

Not applicable

Reviewer Expertise:

Humoral Immunity, Antibody Responses, Follicular T cells

We confirm that we have read this submission and believe that we have an appropriate level of expertise to confirm that it is of an acceptable scientific standard, however we have significant reservations, as outlined above.

# Reviewer report: Reviewer response for version 1

**DOI:** 10.5256/f1000research.150153.r282388

**Published:** 2024-06-10

**Authors:** Philip C Calder

**Affiliations:** 1 University of Southampton, Southampton, England, UK

This manuscript describes the protocol for a human RCT testing 4 doses of tocotrienol rich fraction of palm kernel oil vs placebo using response to influenza vaccination as the main outcome. Other outcomes include some other immune measures and plasma "vitamin E" concentrations. The experimental design is appropriate to test the hypothesis. There is sufficient detail in the protocol. The power calculation is rather unspecific. The trial has been ethically approved and is registered.

 Specific comments:

 1. In section Research question I think "lower dose" should read "different doses"

 2. In section Study hypothesis, again you refer to "low dose", but compared to what? Isn't your hypothesis that "different doses of TRF will have different effects ..".

 3. Ethics considerations, 3rd sentence. You write "changes to" twice.

 4. Sample size. What does "a large difference" mean. To do a sample size calculation you need to define what the "difference" (effect size) will be.

 5. It is not exactly clear how you will randomly assign participants to groups. In an RCT each person should have an equal chance to be allocated to each group. 

 6. Discussion, 2nd sentence. COVID-19 is the disease caused by SARS-CoV-2, it is not the name of the virus, although during the pandemic the two terms unfortunately were often used interchangeably. COVID-19 is coronavirus disease discovered in 2019.

Is the study design appropriate for the research question?

Yes

Is the rationale for, and objectives of, the study clearly described?

Yes

Are sufficient details of the methods provided to allow replication by others?

Yes

Are the datasets clearly presented in a useable and accessible format?

Not applicable

Reviewer Expertise:

Nutritional immunology; human trials

I confirm that I have read this submission and believe that I have an appropriate level of expertise to confirm that it is of an acceptable scientific standard, however I have significant reservations, as outlined above.

# Reviewer report: Reviewer response for version 1

**DOI:** 10.5256/f1000research.150153.r282394

**Published:** 2024-05-30

**Authors:** Sonia Chew Wen Phang

**Affiliations:** 1 Monash University, Sunway, Malaysia

This study aims to investigate the effect of different concentrations (50, 100, 200, 400 mg) of tocotrienol-rich fraction (TRF) on the immune response to an influenza vaccine in healthy participants. Given the heightened awareness of immune health due to the recent pandemic, understanding how nutritional interventions like TRF can enhance immunity is timely and relevant. By utilising both low and high dosages of TRF, the findings of this study may offer a more complete understanding of the biochemical impacts of TRF on the host immune system.

 Overall, the study protocol is concise and clear in its research objectives and study design. The methodology of each approach is explained thoroughly in detail, allowing replication by others.

 Only one point that needs to be addressed as well as a suggestion on terminology:-

 1) Clarification is needed on the age criterion of the participants. In the study plan, it is stated that participants aged between 25 to 60 years old will be invited, while in Table 2, the age range stated in the inclusion criteria is 18 to 60 years old.

 2) The terminology used to refer to the individuals taking part of the study could be standardized for clarity and consistency. I recommend the use of either 'volunteers', 'participants', or 'subjects' consistently throughout the protocol.

Is the study design appropriate for the research question?

Yes

Is the rationale for, and objectives of, the study clearly described?

Yes

Are sufficient details of the methods provided to allow replication by others?

Yes

Are the datasets clearly presented in a useable and accessible format?

Not applicable

Reviewer Expertise:

Biochemistry, diabetes

I confirm that I have read this submission and believe that I have an appropriate level of expertise to confirm that it is of an acceptable scientific standard.
